# The Emergence of Shared Leadership in Innovation Labs

**DOI:** 10.3389/fpsyg.2021.685167

**Published:** 2021-08-12

**Authors:** Robert Rose, Lars Groeger, Katharina Hölzle

**Affiliations:** ^1^Research Group IT-Entrepreneurship, Digital Engineering Faculty, Hasso Plattner Institute, University of Potsdam, Potsdam, Germany; ^2^Macquarie Business School, Macquarie University, Sydney, NSW, Australia; ^3^HPI School of Design Thinking, Potsdam, Germany

**Keywords:** innovation laboratories, intrapreneurship, team creativity, shared leadership, social network analysis

## Abstract

Implementing innovation laboratories to leverage intrapreneurship are an increasingly popular organizational practice. A typical feature in these creative environments are semi-autonomous teams in which multiple members collectively exert leadership influence, thereby challenging traditional command-and-control conceptions of leadership. An extensive body of research on the team-centric concept of shared leadership has recognized the potential for pluralized leadership structures in enhancing team effectiveness; however, little empirical work has been conducted in organizational contexts in which creativity is key. This study set out to explore antecedents of shared leadership and its influence on team creativity in an innovation lab. Building on extant shared leadership and innovation research, we propose antecedents customary to creative teamwork, that is, experimental culture, task reflexivity, and voice. Multisource data were collected from 104 team members and 49 evaluations of 29 coaches nested in 21 teams working in a prototypical innovation lab. We identify factors specific to creative teamwork that facilitate the emergence of shared leadership by providing room for experimentation, encouraging team members to speak up in the creative process, and cultivating a reflective application of entrepreneurial thinking. We provide specific exemplary activities for innovation lab teams to increase levels of shared leadership.

## Introduction

The present-day imperative of innovation has led organizations to embrace intrapreneurship for competitive advantage (Kuratko, 2017; Klofsten et al., 2021). However, intrapreneurs within large organizations are limited in their entrepreneurial endeavors by inflexible rules and routines, hierarchical decision making, and risk-averse leaders (Neessen et al., 2019).

Intrapreneurial collaboration, therefore, is increasingly deployed in so-called innovation laboratories (labs; Caccamo, 2020; Rosenow-Gerhard, 2020; Sund et al., 2021), which have been described as “dedicated physical environments or facilities with collaborative workspaces in which groups and teams of employees can engage with each other in order to explore and extend their creative thinking beyond and above normal boundaries” (Magadley and Birdi, 2009, p. 315), whereas intrapreneurship can be defined as “the practice of developing a new venture within an existing organization, to exploit a new opportunity and create economic value” (Parker, 2011, p. 19). Aligned with the strategy of the parent organization and with access to its resources, innovation labs are set up purposely to nurture a creative, collaborative climate for teams, protected from organizational bureaucracy (Fecher et al., 2020). These labs aim to accelerate the process of entrepreneurial opportunity identification, idea conception to successful new product development, thereby contributing to an organization’s long-term survival.

Extant research suggests that the ability to act entrepreneurially is connected to the flexibility and agility of the organizational culture and its leadership (Schweitzer et al., 2016; Blanka, 2019). Accordingly, a typical feature in innovation lab environments are semi-autonomous, multidisciplinary teams in which multiple team members collectively exert leadership influence (e.g., based on situational demands), thereby challenging traditional command-and-control conceptions of leadership (Groeger et al., 2019). In particular, intrapreneurial teams can be characterized by a high skill differentiation, low authority differentiation, and low temporal stability (Hollenbeck et al., 2012; Knight et al., 2020). The polyphonic and less-hierarchical characteristics of teams engaged in entrepreneurial thinking and doing can presumably engender team creativity but may also make leadership by a single designated individual difficult. Prospective resolutions to the challenges of leading creative teamwork may be found within the discourse on concepts that regard “leadership as a property of the collective” (Cullen-Lester and Yammarino, 2016, p. 173). Among these pluralized leadership perspectives (Denis et al., 2012), the research stream of shared leadership has received substantial attention over the last two decades (see Table 1 for a list of the key literature reviews). However, empirical evidence for the effectiveness of pluralized structures in teams, for which creativity is pivotal, remains limited (e.g., Ali et al., 2020; He et al., 2020). Our research aims to address this gap.

**Table 1:** Key literature reviews on shared leadership.

Article	Review strategy
Wu et al. (2020)	Meta-analytic review of 40 empirical studies
Scott-Young et al. (2019)	Systematic literature review of 104 articles
Sweeney et al. (2019)	Systematic literature review of 40 empirical studies
Zhu et al. (2018)	Narrative review, sample selection not specified
D’Innocenzo et al. (2016)	Meta-analytic review of 43 empirical studies
Barnett and Weidenfeller (2016)	Narrative review of 72 articles
Dust and Ziegert (2016)	Systematic literature review of 175 articles
Nicolaides et al. (2014)	Meta-analytic review of 52 empirical studies
Wang et al. (2014)	Meta-analytic review of 40 empirical studies
Denis et al. (2012)	Narrative review, incl. 19 articles on shared leadership
Yammarino et al. (2012)	Narrative review, sample selection not specified
Friedrich et al. (2009)	Narrative review, sample selection not specified

*We also included articles where the review scope includes other forms of plural leadership if a substantial portion was dedicated to the concept of shared leadership. We excluded book chapters for brevity (e.g., Friedrich and Zhong, 2017; Wassenaar and Pearce, 2018)*.

While research on the various forms of innovation labs (e.g., digital labs, idea labs, and accelerators) is mainly focused on the broader outcomes of entrepreneurship and innovation from a strategic perspective (e.g., Lewis and Moultrie, 2005), limited research explored the human side of these work environments (e.g., Fecher et al., 2020), the intrapreneurial team member’s networks and coordination. At the same time, the distinct context of innovation labs has been largely overlooked in teams research despite the acknowledgment that effective teamwork is a critical element of innovation lab performance (Osorio et al., 2019). Horizontal coordination of activities requires team members to share leadership behaviors in lieu of a formal, vertical team leader. A crucial question for teams in the context of innovation labs, therefore, is the effectiveness of their shared leadership structure and which team factors contribute to the emergence of such.

Consequently, we examine potential antecedents for the emergence of shared leadership in teams operating in an innovation lab environment. Proposed antecedents include a team’s experimental culture, task reflexivity, and voice. Further, we evaluate the influence of shared leadership on a team’s creative output. We follow a social network approach and adopt an importance-weighted density (IWD) operationalization of shared leadership (Lemoine et al., 2020). We identify factors specific to creative teamwork that facilitate the emergence of shared leadership by providing room for experimentation, encouraging team members to speak up in the creative process, and cultivating a reflective application of entrepreneurial thinking. Further, we provide specific exemplary activities for innovation lab teams to increase levels of shared leadership.

Increasingly popular innovation labs represent a setting in which shared leadership in teams coincides with the application of collaborative intrapreneurial approaches. With this study, we contribute to the research stream of team effectiveness and creativity in innovation labs, presenting a more nuanced and deeper understanding of teams’ functioning and leadership as they strive for innovation in a non-traditional, intrapreneurial context.

## Hypothesis Development

In this section, we first outline the concept of shared leadership. Second, we submit potential antecedents of shared leadership in a creative context, and third, we describe the relationship of shared leadership and team creativity.

### Shared Leadership

Whereas the focus in leadership research has traditionally been the influence of a single, designated leader (Meuser et al., 2016), the inquiry of plural forms of leadership has gained considerable momentum over the last decade (Denis et al., 2012; Ospina et al., 2020). A multitude of available perspectives (e.g., distributed, collaborative, collective, emergent, network, rotating, or shared leadership) view leadership as a collective phenomenon (Cullen-Lester and Yammarino, 2016). At the team level, the prevalent concept under study has been shared leadership, defined by Carson et al. (2007, p. 1218) as “an emergent team property that results from the distribution of leadership influence across multiple team members”. Among team-centric leadership concepts (Kozlowski et al., 2016; van Knippenberg, 2017), shared leadership can be categorized using a framework offered by Morgeson et al. (2010), who differentiate the source of team leadership by its locus and formality. Accordingly, shared leadership constitutes an informal and internal source of team leadership, also described as a “non-traditional” configuration of leadership structure (Morgeson et al., 2010, p. 28).

Although multiple literature reviews point out the discordant conceptualizations of shared leadership (see Table 1), available meta-analytic findings consistently show incremental effects of shared leadership on team effectiveness dimensions “above and beyond vertical leadership” (Wang et al., 2014, p. 193). Notwithstanding, scholars widely acknowledge the interdependence of shared leadership and vertical forms of leadership for effective teamwork (e.g., D’Innocenzo et al., 2016). Table 1 provides an overview of the key literature reviews on shared leadership.

Theoretical considerations for the phenomenon of innovation labs, and particularly, the role of team management and leadership in these novel organizational entities, are at a nascent stage. In scholarly literature, Lewis and Moultrie (2005) coined the term “innovation laboratory.” A conceptual framework introduced by Moultrie et al. (2007), recently iterated by Osorio et al. (2019), mentions teamwork as a key element of innovation lab performance. In the same vein, Caccamo (2020) and Sund et al. (2021) regard teams as the focal work unit in innovation labs. More specifically, Fecher et al. (2020) connected teams research with the innovation lab literature and call to further “examine participants’ perceptions toward specific team dynamics” in the future research (Fecher et al., 2020, p. 574). Notwithstanding the valuable insights that can be drawn from case-based studies on innovation lab management, theory and evidence on the leadership of teams in the novel context of innovation labs are missing. However, research on team creativity and innovation regards leadership as a key ingredient of effective teamwork (Paulus and Kenworthy, 2018), and we draw from recent works on shared leadership in particular to hypothesize antecedents in the following.

### Antecedents of Shared Leadership in Creative Contexts

Following the seminal study of Carson et al. (2007), a wealth of subsequent empirical work tested further antecedent conditions that explain the emergence of shared leadership. Recent integrative reviews (Wassenaar and Pearce, 2018; Zhu et al., 2018; Sweeney et al., 2019) summarized antecedents generally in categories – vertical leadership and the external environment (e.g., empowering leadership and support structures) or team (member) characteristics and the internal environment (e.g., team diversities and task-related factors).

Empirical research on antecedents of shared leadership specific to teamwork in creative and intrapreneurial contexts, however, is notably rare. Serban and Roberts (2016) confirmed the positive influence of the internal team environment (Carson et al., 2007) and identified task ambiguity and cohesion as further predictors of shared leadership based on a sample of student teams working on a creative task. Hoch (2013) reported, for a sample of organizational work teams, a positive influence of team member integrity and vertical leadership on shared leadership, which in turn was found to be a predictor of innovative work behavior of team members. Other studies focusing on the relationship of team creativity and shared leadership proposed the latter as the focal antecedent, on which we elaborate on in our section for team creativity.

#### Experimental Culture

Although conceptually related to participative or psychological safety (Edmondson and Lei, 2014) and the general domain of team climate constructs, the experimental culture of a team is distinctly defined as “a culture that provides room for experimentation and is tolerant of ‘competent’ mistakes” (Vera and Crossan, 2005, p. 207). This tolerance for failure has been emphasized as a key attribute of intrapreneurs (Neessen et al., 2019), teamwork in innovation or idea labs (Narayanan, 2017), and represents a culture necessary for organizations engaging in experimentation and intrapreneurship (Hampel et al., 2020). Experimental culture can be viewed as a state of “nonjudgmental inquiry,” which (Raelin, 2006) suggested as a prerequisite for collaborative forms of leadership. Arguably more so than with other knowledge-intensive tasks, the sharing of leadership in creative teamwork benefits from individual team members enacting leadership influence in potentially uncomfortable situations or, put differently, it requires a culture of “yes-anding” (Hadida and Tarvainen, 2015). We assert that an experimental culture does not just facilitate team improvisation (Vera and Crossan, 2005), it further enables members to participate in the sharing of leadership in creative teamwork, and posit as:

H1: A team’s experimental culture is positively related to its level of shared leadership.

#### Task Reflexivity

Reflexivity in teams is defined as “the extent to which team members overtly reflect upon the group’s objectives, strategies, and processes and adapt them to current or anticipated endogenous or environmental circumstances” (West, 1996, p. 559). Higher levels of team reflexivity have been consistently identified as a beneficial determinant for entrepreneurial efforts (Knipfer et al., 2018; Xiong, 2020). Task reflexivity, in particular, has been emphasized for its relevance in creative, innovation-driven teamwork that revolves around complex challenges (Tjosvold et al., 2004; Chen et al., 2019) and empirical evidence supports the notion of a positive influence of reflexivity on creative, innovation-driven teamwork (for a recent review see, Schippers et al., 2017).

Tasks of high interdependence, complexity, and creativity have been proposed as eligible for shared team leadership (Pearce, 2004). These types of tasks also commonly characterize team-based entrepreneurial decision making (Patzelt et al., 2020). Consequently, we argue that the better members of a team can reflect upon complex tasks in creativity-focused teamwork, the better they can enact leadership behaviors apposite to individual talent and situational demands (Aime et al., 2014), and posit as:

H2: A team’s task reflexivity is positively related to its level of shared leadership.

#### Voice

Freedom to experiment and reflect together upon complex tasks presumably sets the stage for the emergence of shared leadership. However, eventually, team members should also feel encouraged “to speak up and get involved,” as asserted by Carson et al. (2007, p. 1223). The authors introduced the higher-order factor of internal team environment, which has found repeated empirical support to predict levels of shared leadership (Carson et al., 2007; Daspit et al., 2013; Serban and Roberts, 2016). This conceptualization has also received criticism due to a “conflation of intragroup environments and behaviors” (Paunova, 2015, p. 947) – that is, among the three lower-order dimensions of shared purpose, social support, and voice (Carson et al., 2007). However, our present focus on teamwork in creative environments implicates voice as a focal construct, as it reflects a necessary condition in iterative innovation processes to actively articulate input on how complex challenges should be approached (Kremer et al., 2019). Based on the broad stream of research on (employee) voice (as recently reviewed by Bashshur and Oc, 2014; Mowbray et al., 2015), we adopt a conceptualization proposed by Morrison (2011, p. 375), who defines voice as a “discretionary communication of ideas, suggestions, concerns, or opinions about work-related issues with the intent to improve organizational or unit functioning”. Beyond the established positive relationship of voice with creativity and innovation (e.g., Liang et al., 2019; Ali et al., 2020) and by following recent calls to account for voice in entrepreneurial teams (Patzelt et al., 2020), we contend that giving team members the opportunity for voice in creative teamwork (Friedrich and Zhong, 2017) facilitates the emergence of shared leadership and posit as:

H3: Voice is positively related to a team’s level of shared leadership.

#### Team Creativity

Outcome-oriented creativity is commonly defined following the works of Teresa M. Amabile and colleagues as “the production of novel and useful ideas by an individual or small group of individuals working together” (Amabile, 1988, p. 126; Amabile and Pratt, 2016, p. 158). This raises the obvious question of how these efforts can be led effectively. The relationship of leadership and creativity has consequently received ample attention over the last decades (for recent reviews see Mainemelis et al., 2015; Lee et al., 2020). Despite repeated calls to investigate the particular influence of shared leadership on team creativity (e.g., Gilson et al., 2015; Friedrich and Zhong, 2017), empirical evidence on this very relationship remains scant. He et al. (2020) surveyed undergraduate teams and corporate research and development teams and found the influence of shared leadership on team creativity to be mediated by individual creativity and self-efficacy. They also identified a moderating influence of transformational leadership by a designated team leader. Empirical evidence for a direct positive influence of shared leadership on team creativity was found for undergraduate student teams (Lee et al., 2015; Sun et al., 2016), organizational teams (Ali et al., 2020; Cavazotte and de Paula, 2020; Song et al., 2020), and inter-organizational teams (Gu et al., 2018). Aime et al. (2014, p. 328) confirmed this positive influence on team creativity for the related concept of “power heterarchy” based on a sample of cross-functional student teams, stating that less-hierarchical teams are enabled “to leverage the diverse and unique capabilities of individual members”. In line with the previous research, we therefore argue that teams tasked with creative work benefit from higher levels of shared leadership and posit as:

H4: A team’s level of shared leadership is positively related to the creativity of its output.

## Materials and Methods

### Sample and Procedure

The teams of our sample are part of an innovation lab program for graduates at a higher educational institution in Germany. The training involves two consecutive terms structured by team-based projects of three-, six-, and twelve-weeks tenure. Participants of our study were either part of a six-week project team (initial term) or a twelve-week project team (consecutive term) and therefore were experienced in creative teamwork. The graduates we surveyed account for a population of interest (Stevens, 2011) for innovation lab practice as they are part of the future workforce and thus, are adequate to learn about the emergence of shared leadership in intrapreneurial teams. Teams of the program are provided with innovation-focused challenges from external project partners and are co-located in one space; they are supported by the same administration and coaching staff. The physical space of this institution is widely considered a prototypical version of an innovation lab and has been repeatedly mimicked by western-based, large corporates that use this environment for their employee creativity training.

Each team is assigned one to three team coaches. The team leadership configurations of our sample can be characterized as emergent, since team coaches primarily provide method- and process-focused guidance from outside – the teams do not feature designated leaders. Consequently, teams work with a high degree of autonomy by design, operating in a structured environment, and are required to organize their work within their teams themselves. The assigned team coaches and team memberships were stable throughout the project for each team.

Before approaching the teams, we discussed specifics of the study with the program staff (e.g., to check for potentially unclear instructions or confronting questions). We then administered the survey close to the end of the program, prior to the conclusion of all projects, to allow for a mental aggregation focused on the current team project by members and coaches, respectively. After introducing our study to all participants and coaches onsite in one session, we asked for voluntary participation and distributed paper-and-pencil questionnaires. Several coaches accompanied more than one team and provided evaluations for each of them.

We collected complete questionnaires from 104 team members and 49 evaluations from 29 coaches. This resulted in a response rate of 92% of the full program and a final sample of 21 teams. Team sizes ranged from three to six members (*M* = 4.95, *SD* = 0.74) and teams were accompanied by 2.33 coaches on average (*SD* = 0.73). Among team members, the mean age was 28.86 years (*SD* = 2.75) and 54.8% were female. Among team coaches, the mean age was 35.1 years (*SD* = 5.54) and 58.7% were female. The sample included 18 different nationalities in terms of citizenship, with 79.8% of team members and 89.7% of team coaches being German citizens.

### Measures

Our study variables – experimental culture, task reflexivity, and voice – were measured using a seven-point Likert scale ranging from 1 (strongly disagree) to 7 (strongly agree). All items were provided in English, which is the working language across the incubation program. Instructions referred to the team unit except for individually assessed control variables. Team members responded with their perceptions of experimental culture, task reflexivity, voice, and shared leadership. Coaches evaluated their teams’ degree of creativity based on the prototypes developed for the assigned project challenge. See Table 2 for our complete list of measures. Program staff provided us with additional archival data, such as the assigned coaches.

**Table 2:** List of measures.

**Shared leadership** Carson et al. (2007)
To what degree does your team rely on this individual for leadership?
**Team creativity** [**adapted from** Yong et al. (2014)]
The final concept contains an interesting insight/idea that is not derivative of existing work
The final concept uses new ideas to solve old problems more effectively than existing ideas
The final concept addresses an important aspect of the challenge with the use of new ideas
Overall, the final concept is very original
Overall, the final concept is very novel
Overall, the final concept is very different
The final concept has an appealing simplicity (i.e., it is not overly complex)
The final concept is theoretically feasible
The final concept is feasible from a practical standpoint
The ideas and approach used in the final concept are relevant to the team’s given challenge
Overall, the final concept has valuable practical implications
**Experimental culture** Vera and Crossan (2005)
In our team errors are considered as a source of learning
In our team there is room for initiative
In our team there is freedom to experiment
We encourage each other to take risks when trying new ideas
**Task reflexivity** [**adapted from** Tjosvold et al. (2004)]
We as a team regularly discuss whether we are working together effectively
The methods used by our team to get the job done are often discussed
In our team, we modify our objectives in the light of changing circumstances
We often discuss how well we communicate with each other
We as a team often review our approach to getting tasks done
Members of our team identify strengths in their work and areas that need improvement
We as a team are committed to ongoing improvement
We as a team are open to improved ways of working
**Voice** Carson et al. (2007)
The members of our team are encouraged to speak up in discussions
As a member of this team, I have a real say in how we carry out our work
Everyone in our team has a chance to participate and provide input
Our team supports everyone actively participating in decision making

#### Shared Leadership

According to D’Innocenzo et al. (2016), the measurement practice of shared leadership can be categorized into two common approaches. Studies that follow aggregate conceptualizations require respondents to self-report their perceptions of leadership for the team as a whole, which represents a referent-shift composition procedure (Chan, 1998). Alternatively, social network conceptualizations use round-robin ratings by providing each respondent with a list of their respective peers, who are then rated for their individual leadership influence within the team, either overall or specific to lower-order dimensions.

Limitations of the former aggregate approach, which have been repeatedly identified by reviews (e.g., Gockel and Werth, 2010; D’Innocenzo et al., 2016; Zhu et al., 2018), involve the undifferentiated conceptualization of shared leadership and the adoption of established leadership scales (e.g., transformational). These were initially developed to assess the perception of leadership behaviors of individuals. By contrast, social network approaches allow for the examination of relational structures of leadership influence in teams (Park et al., 2020), consonant with shared leadership theory (Mayo et al., 2003; Carter et al., 2015). Meta-analytic findings have further shown that the network measurement approach of shared leadership results in higher effect sizes (Nicolaides et al., 2014; D’Innocenzo et al., 2016; Wu et al., 2020).

Network properties at the team level can be indexed by density, indicating the extent of displayed shared leadership behaviors of a team (e.g., Carson et al., 2007), or by decentralization, reflecting the distribution of shared leadership (e.g., Small and Rentsch, 2010). Most recently, Lemoine et al. (2020) observed that both network measures had been often used and interpreted interchangeably in shared leadership research, although they depict distinct network characteristics; thus, they are indicators of varying conceptions of the shared leadership construct (Lemoine et al., 2020). As a potential remedy, the authors propose the alternative network index of IWD, which “takes into account the magnitude of a node’s incoming ties, the relative centrality of that node compared to others, as well as the relative influence and centrality of contacts from whom ties emerge” (Lemoine et al., 2020, p. 440). Informed by the rationales of density, decentralization, and eigenvector centralization, the IWD allows for a conceptualization of shared leadership that adequately reflects its theoretical nature.

We follow the social network approach and adopt the IWD operationalization of shared leadership to respond to calls for increased methodological diversity in creativity and innovation research (Rose et al., 2020). By employing a valued round-robin rating among team members, we asked the widely used question from Carson et al. (2007): “To what degree does your team rely on this individual for leadership?” on a scale from 1 (not at all) to 7 (to a very great extent). The IWD was computed using the network calculator provided by Lemoine et al. (2020).

#### Team Creativity

Each team’s coaches evaluated team creativity in terms of novelty and usefulness regarding the final concept prototyped by the team for its innovation challenge. Although our measurement approach does not qualify for a time-lagged design, the evaluation of concepts – instead of individual creativity or more team-centric creativity measures (e.g., Jiang and Zhang, 2014) – introduces an element of temporal separation to our study (Podsakoff et al., 2012; Spector, 2019) because the teams developed their concepts over the course of the whole project. We used a scale developed by Yong et al. (2014), which has been validated in a similar empirical setting of multidisciplinary teams of graduates. One context-specific item was dropped, and we slightly adapted the remaining 11 items to fit our research context. A sample item was “The final concept is feasible from a practical standpoint.” Cronbach’s alpha for our sample was 0.9.

#### Experimental Culture

Team members were asked to describe their team’s experimental culture using a four-item scale developed by Vera and Crossan (2005). A sample item was “In our team there is freedom to experiment.” Cronbach’s alpha for our sample was 0.75.

#### Task Reflexivity

Team members assessed their team’s task reflexivity through a scale from Tjosvold et al. (2004), of which we dropped one item and slightly adapted phrasings to account for the present research setting. Our sample’s Cronbach’s alpha for the eight-item scale was 0.77.

#### Voice

Team members’ perception of voice was captured using a four-item scale from Carson et al. (2007), which was developed based on works from De Dreu and West (2001) and Van Dyne and LePine (1998). Cronbach’s alpha for our sample was 0.73.

#### Statistical Control

Each team of the program worked in the same quasi-prototypical innovation lab setting, which simplified the consideration of context and statistical control. A preliminary analysis indicated no significant differences among the six- or twelve-week projects. Further, teams were purposefully staffed by program managers to ensure equally diverse teams across the program. To check for the intended team compositions, we calculated diversities in terms of variety using Harrison and Klein’s (2007) corrected version of Blau (1977) index, as recommended by Biemann and Kearney (2009) for the case of varying team sizes. Relatively low standard deviations among the resulting team values confirm the intentional staffing strategy regarding diversities (Blau_N_; diversity indices not comparable) for gender (*M* = 0.60; *SD* = 0.04), nationality (*M* = 0.38; *SD* = 0.21), and educational background (*M* = 0.83; *SD* = 0.18).

A notable feature among our sampled teams is the mean age of team members. The surveyed program is neither integrated nor synchronized with curricula of nearby universities or companies. Thus, graduates can decide to participate at any point in their studies or work with team members of varying ages. Following recommendations for the consideration of statistical control (Becker et al., 2016), we integrated mean team age as our control variable to check for potential alternative explanations since mean age was previously found to influence shared leadership (Müthel et al., 2012).

### Data Aggregation and Analytic Strategy

As hypothesized, we specified all study variables at the team level. The variables self-reported by team members were aggregated according to the referent-shift consensus model (Chan, 1998). To justify our aggregation procedure, we consulted indices for interrater agreement and interrater reliability (LeBreton and Senter, 2008) and computed estimates using a tool provided by Biemann et al. (2012), reported in Table 3. For interrater agreement, estimates for the mean within-group agreement index r_wg(J)_ (James et al., 1984) ranged from 0.83 to 0.90, indicating “strong agreement” (LeBreton and Senter, 2008) with support for aggregation. For interrater reliability, estimates for the intraclass correlation coefficients ranged from small to medium effects (LeBreton and Senter, 2008). Following recommendations by Woehr et al. (2015) for the interpretation of ICC values, we could justify aggregation given similarly low estimates for the previous scale applications (e.g., for task reflexivity, see De Dreu, 2007; for voice, see Serban and Roberts, 2016).

**Table 3:** Indices for interrater agreement and interrater reliability.

Variable	Interrater agreement		Interrater reliability			
	*Mean of r_wg(J)_*	*SD of r_wg(J)_*	*F ratio*	*p*	*ICC(1)*	*ICC(2)*
Experimental culture	0.86	0.10	1.87	0.03	0.15	0.47
Task reflexivity	0.90	0.06	2.03	0.01	0.17	0.51
Voice	0.83	0.21	1.42	0.14	0.08	0.30

Estimates of *r_wg(J)_* were calculated based on the uniform null distribution ($\sigma_{EU}^{2}$ = 4).

To test the veracity of our hypothesized relationships, we performed hierarchical regression analyses. Given our limited sample size, we conducted separate regression analyses for each predictor by entering control first, and then the main predictor of interest, testing hypotheses independently to maintain statistical power (Cohen et al., 2003). This is in line with the testing strategies of comparable research models (e.g., Vera and Crossan, 2005; Carson et al., 2007; Serban and Roberts, 2016) or team studies with similar sample size restrictions (e.g., Wu and Cormican, 2016; Uitdewilligen and Waller, 2018). The resulting separate measurement models did not imply further soundness verification, such as robustness tests for potential misspecification. Instrument reliabilities can be considered appropriate based on Cronbach’s alphas above the disputed but common threshold of 0.70 (e.g., Greco et al., 2018). We accounted for method bias (Podsakoff et al., 2012; Spector, 2019) insofar as predictor variables and the criterion variable were rated by different groups, that is, teams and coaches, respectively.

## Results

We hypothesized first that experimental culture, task reflexivity, and voice are positively associated with shared leadership (Model 1), and second, that shared leadership is positively associated with coach-rated team creativity (Model 2). Table 4 presents team-level means, standard deviations, and correlations among our focal variables. Given the limited sample size, we can cautiously interpret the positive correlations as preliminary support for our hypotheses. Notably, the mean age of team members correlates negatively with shared leadership at a statistically significant level (*p* < 0.01).

**Table 4:** Team-level means, standard deviations, and correlations.

Sl. No.	Variable	*M*	*SD*	1	2	3	4	5	6
1.	Mean team age	28.90	1.11	–					
2.	Experimental culture	5.25	0.56	−0.02	–				
3.	Task reflexivity	5.13	0.51	−0.09	0.58^**^	–			
4.	Voice	5.70	0.52	−0.02	0.68^***^	0.55^**^	–		
5.	Shared leadership	0.48	0.12	−0.53^**^	0.36^†^	0.47^*^	0.36^†^	–	
6.	Team creativity	5.39	0.86	0.30^†^	0.37^*^	−0.05	0.06	−0.24	–

Indicated significance at

† *p* < 0.10;

* *p* < 0.05;

** *p* < 0.01;

*** *p* < 0.001.

Sample of *n* = 21 teams (104 students). Team creativity was rated through 49 evaluations of 29 team coaches.

Table 5 displays the results of our regression analyses, testing Hypotheses 1–4. First, we posited that a team’s experimental culture is positively related to shared leadership. While the zero-order correlation with shared leadership was significant at a mere relaxed level (*p* < 0.10), we found partial support for Hypothesis 1 in regression Model 1a (adj. *R*^2^ = 0.34, *p* < 0.01) when controlling for mean team age. Experimental culture proved the only focal variable significantly correlated with coach-rated team creativity (*r* = 0.37, *p* < 0.05). Second, Hypothesis 2 states that a team’s ability to reflect upon its application of the creative problem-solving process positively influences shared leadership. Model 1b supported this proposed relationship (adj. *R*^2^ = 0.40, *p* < 0.01). Task reflexivity also shows the highest statistically significant correlation with shared leadership among our focal variables (*r* = 0.47, *p* < 0.05). Third, Hypothesis 3 predicted a positive influence of voice on shared leadership, which is supported by Model 1c (adj. *R*^2^ = 0.34, *p* < 0.01). Voice further exhibits a strong and statistically significant correlation with a team’s experimental culture (*r* = 0.68, *p* < 0.001). Fourth, for Hypothesis 4, which predicted positive association of shared leadership and team creativity, we could not detect a statistically significant influence of shared leadership on team creativity in Model 2. Finally, statistical control by the mean age of team members had a statistically significant influence in the analyzed models. The negative regression coefficients (*B* < −0.05, *p* < 0.05) indicate alternative explanations for the explained variance since teams with younger members, on average, showed higher levels of shared leadership with the proposed main predictors.

**Table 5:** Results of regression analyses.

	*B*	*SE*	*R*^2^ (adj. *R*^2^)	*∆R* ^2^	*F*
Step/Model/Independent variables	Dependent variable: Shared leadership				
*1st Step of Model 1: Control*	−0.06^*^	(0.02)	0.28 (0.24)		7.35^*^
*2nd Step of Model 1*					
Model 1a:			0.40 (0.34)	0.12	6.05^**^
Control	−0.06^*^	(0.02)			
Experimental culture	0.08^†^	(0.04)			
Model 1b:			0.46 (0.40)	0.18	7.67^**^
Control	−0.05^*^	(0.02)			
Task reflexivity	0.10^*^	(0.04)			
Model 1c:			0.40 (0.34)	0.12	6.04^**^
Control	−0.06^*^	(0.02)			
Voice	0.08^†^	(0.04)			
	**Dependent variable: Team creativity**				
*1st Step of Model 2: Control*	0.23	(0.17)	0.09 (0.04)		1.92
*2nd Step of Model 2*			0.10 (0.00)	0.01	1.01
Control	0.19	(0.20)			
Shared leadership	−0.79	(1.89)			

Unstandardized regression coefficients are reported.

† *p* < 0.10;

* *p* < 0.05;

** *p* < 0.01

In balance, the proposed antecedents could explain a relatively medium amount of variance in shared leadership when controlling for mean team age with statistically significant regression model results (*p* < 0.01). Therefore, Hypotheses 1–3 (experimental culture, task reflexivity, and voice) found empirical support, but we could not verify Hypothesis 4 (team creativity).

## Discussion

Our study set out to explore the emergence of shared leadership in a creative context based on the literature of teams research and innovation studies. We surveyed 104 students and 29 coaches nested in 21 teams working in a prototypical innovation lab environment. Our results provide partial empirical support for a positive influence of a team’s experimental culture, task reflexivity, and voice on the IWD of shared leadership when controlling for mean team age. We could not confirm a hypothesized positive relationship between shared leadership and team creativity.

### Contribution

By following calls to examine plural forms of leadership in creative environments (Friedrich and Zhong, 2017), our study offers the following contributions to theory and practice.

First, our team-centric perspective on innovation laboratories can inform the evidence-based management of this increasingly implemented form of present-day intrapreneurial efforts. We identified factors specific to creative teamwork that facilitate the emergence of shared leadership, as measured by social network indicators. Beyond the previously established link of a team’s experimental culture and its ability to improvise (Vera and Crossan, 2005), our findings demonstrate a positive relationship with shared leadership. Experimental culture in knowledge-intensive contexts represents a “latitude to be spontaneous and to take risks and make mistakes” (Krylova et al., 2016, p. 1053). To acknowledge the possibility of failure and to try out the unusual when team members become involved in shared leadership is also indicative of a culture conducive to intrapreneurship (e.g., Elert and Stenkula, 2020).

Second, our results further show a positive association between shared leadership and the collective task reflection of the team in line with the emphasis on reflexivity for creative teamwork (Paulus and Kenworthy, 2018). We also confirmed the perceived opportunity for voice in a team as an antecedent of shared leadership, similar to the previous empirical works (Carson et al., 2007; Daspit et al., 2013; Serban and Roberts, 2016). While experimental culture is “setting the stage” for a failure-tolerant involvement in shared leadership, voice implies the perceived opportunity for active participation by “strengthening both a common sense of direction and the potential for positive interpersonal support” (Carson et al., 2007, p. 1223). Voice can be fostered by open feedback and establishing developmental performance management systems (Kremer et al., 2019) in the context of innovation lab management (Osorio et al., 2019).

Third, and lastly, we could not confirm the hypothesized positive influence of shared leadership on team creativity. One potential explanation can be found in Aime et al. (2014). The authors’ findings show that shifts of power to members with the required expertise for a given situational demand increases team creativity; however, this only occurs if team members attribute legitimacy to power shifts within the team (Aime et al., 2014), a condition we did not consider in our study. A further contingent factor beyond the scope of this study involves the creative efficacy of the team and its members, that is, the “team’s confidence in its abilities and efforts in utilizing team resources to successfully generate creative outputs” (Ali et al., 2020, p. 410). In addition, the translation of shared leadership to creative team outputs can also be influenced by expert coaches from outside the team as a form of functional leadership (Morgeson et al., 2010) which we account for in the limitations of our study.

### Managerial Implications for Innovation Labs

In all, this study bears relevance for the management of innovation labs insofar as our presented findings implicate the opportunity to facilitate the emergence of team shared leadership in a creative context. The team-centric nature inherent to the many incarnations of increasingly popular innovation labs (e.g., intrapreneurial support structures, such as digital labs, accelerators, and incubators) calls for more evidence-informed insights on how present-day teams work in these novel contexts (Ahuja, 2019). In particular, the collective engagement in task- and situation-specific intrapreneurial activities requires team members to share leadership behaviors in lieu of a formal, vertical team leader. Innovation lab and team managers, therefore, should encourage a team culture that allows for the emergence of shared leadership to let teams’ maneuver’ intrapreneurial activities with increased autonomy in a context supportive to innovation (Van de Ven, 2017). This can be achieved by providing room for experimentation, encouraging team members to speak up in the process, and cultivating a reflective application of entrepreneurial thinking. In the following Table 6, we build upon and extend previous work in entrepreneurship education (Groeger and Schweitzer, 2020) to make suggestions about commendable objectives associated with learning about antecedents of shared leadership and describe how these objectives could be facilitated via specific learning and coaching activities. We propose this as a starting point toward better managing teams in innovation lab settings.

**Table 6:** Objectives and activities for shared leadership antecedents.

Shared leadership antecedents	Commendable objectives (*What* should innovation lab teams learn?)	Commendable activities (*How* to facilitate behaviors?)
*Experimental culture* – “a culture that provides room for experimentation and is tolerant of ‘competent’ mistakes” Vera and Crossan (2005, p. 207)	Increase tolerance for failure, learning by “informed trial and error” Emphasizing exploration over exploitation when beginning an iteration of the creative teamwork process	Practice creating experiments to test assumptions and gather facts, encouraging an iterative approach through multiple rounds of short and intensive time-boxed sessions Encouraging action, improvisation, and play at work to experience how iterative processing of information affects perceptions of uncertainty Practice yes-anding (“Agree, Accept, and Add”) e.g., in brainwriting sessions Continuously involve potential users in prototyping and testing of solutions Allow for temporary team membership changes among the innovation lab teams to foster impartiality and knowledge exchange Provide changing inspirational resources and an abundance of stationary supplies to the teams on site
*Task reflexivity* – “the extent to which team members overtly reflect upon the group’s objectives, strategies, and processes and adapt them to current or anticipated endogenous or environmental circumstances” West (1996, 559)	Create a shared vision for the team and project Apply team formation, management, and conflict resolution approaches Appreciate and integrate team members’ diverse contributions to develop team-specific competencies Lacerenza et al. (2018) Appreciate the value of ongoing improvement, develop a collective mindset that anticipates inevitable adaption	Share and reflect upon individual preferences about “*how*” to work in team-based contexts Set SMART team goals and revisit regularly; collaboratively design a common vision and refer to it during regular team check-ins Turn assumptions and opinions into testable hypotheses and collect evidence to confirm or reject assumption Reconsider the set of (digital) tools used for communication, ideation, and process documentation when new members join the team
*Voice* – “discretionary communication of ideas, suggestions, concerns, or opinions about work-related issues with the intent to improve organizational or unit functioning” Morrison (2011, p. 375)	Develop group norms, e.g., by establishing respectful and polite team communication Prioritize and reinforce knowledge sharing and integration Balance promotive voice (i.e., “members’ expression of new ideas or suggestions that deviate from the status quo,” Liang et al. (2019, p. 92) and prohibitive voice (i.e., “members’ expression of concerns about possible problems in the team’s approach” Liang et al. (2019, p. 92) in the team	Follow a “guide on the side” coaching approach teams to facilitate team interaction to give everyone a voice; define and rotate team roles Conduct regular feedback sessions within teams, ideally externally facilitated Instill a sense of constant curiosity among team members, provide time and space to explore surprising facts and opinions and share among team members Be mindful regarding the timing for team development interventions, e.g., by coaches

*Source: Modified and extended based upon Groeger and Schweitzer (2020, p. 66)*.

### Limitations and Future Research

Our findings should be interpreted against several limitations of this study, which also indicate foci for the future research. The main objective of our article was to examine the emergence of shared leadership in an innovation lab environment at the team level, which implied an empirical field setting. Therefore, our study is subject to a limited sample size common in field-based teams research in very specific performance environments (Bell et al., 2018). Future empirical endeavors could remedy this restriction using alternative research approaches, such as experience sampling or video-based measurements while alternative empirical strategies would also allow for research of temporal dynamics of emergent team phenomena (Kozlowski, 2015). We conceptualized shared leadership in line with the previous social network approaches (Carter et al., 2015); however, our cross-sectional research design did not allow us to examine how team shared leadership networks evolve over time. Third, in line with the previous shared leadership research using social network measures (Park et al., 2020), we refrained from guiding participants by providing a specific definition of leadership. As varying perceptions of leadership can result in meaningful differences of round-robin ratings for the emergence of shared leadership, the future studies could remedy for this by asking for specific leadership behaviors (Hanna et al., 2021). Fourth, we considered the role of professional coaches to be focused on guidance for creative teamwork. Building on the previous shared leadership research that emphasizes interactions with vertical leader influences, it could be presumed that team coaching can also shape the emergence or effectiveness of shared leadership. Still, the concept of team coaching is highly specific to the respective empirical context and has been understood in distinct ways (Jones et al., 2019).

### Conclusion

Thus far, research on the phenomenon of shared leadership has provided few insights on how it materializes in creative environments. Increasingly, popular innovation labs represent a setting in which shared leadership in teams coincides with the application of a collaborative creative problem-solving approach. Building on extant shared leadership and innovation research, we proposed potential antecedents customary to creative teamwork. Our results demonstrate that experimental culture, task reflexivity, and voice are positively associated with shared leadership. Further research on potential contextual conditions using larger samples is required to investigate the influence of shared leadership on team creativity. In conclusion, this study can inform the management of team-based innovation labs by suggesting how to facilitate shared leadership, specifically in creative teamwork, where people are at the heart of successful innovation initiatives.

## Data Availability Statement

The raw data supporting the conclusions of this article will be made available by the authors, without undue reservation.

## Ethics Statement

Ethical review and approval was not required for the study on human participants in accordance with the local legislation and institutional requirements. The patients/participants provided their written informed consent to participate in this study.

## Author Contributions

RR, LG and KH contributed to conception and design of the study. RR organized the collection of field data, supported at times by LG, performed the statistical analysis, and wrote the first draft of the manuscript. LG and KH wrote sections of the manuscript. All authors contributed to manuscript revision, read, and approved the submitted version.

## Conflict of Interest

The authors declare that the research was conducted in the absence of any commercial or financial relationships that could be construed as a potential conflict of interest.

## Publisher’s Note

All claims expressed in this article are solely those of the authors and do not necessarily represent those of their affiliated organizations, or those of the publisher, the editors and the reviewers. Any product that may be evaluated in this article, or claim that may be made by its manufacturer, is not guaranteed or endorsed by the publisher.
